# Molecular Mechanisms and Therapeutic Strategies for Levodopa-Induced Dyskinesia in Parkinson’s Disease: A Perspective Through Preclinical and Clinical Evidence

**DOI:** 10.3389/fphar.2022.805388

**Published:** 2022-04-07

**Authors:** Ritam Bandopadhyay, Nainshi Mishra, Ruhi Rana, Gagandeep Kaur, Mohammed M. Ghoneim, Sultan Alshehri, Gulam Mustafa, Javed Ahmad, Nabil. A. Alhakamy, Awanish Mishra

**Affiliations:** ^1^ Department of Pharmacology, School of Pharmaceutical Sciences, Lovely Professional University, Phagwara, India; ^2^ Department of Pharmacy Practice, College of Pharmacy, AlMaarefa University, Ad Diriyah, Saudi Arabia; ^3^ Department of Pharmaceutics, College of Pharmacy, King Saud University, Riyadh, Saudi Arabia; ^4^ College of Pharmacy (Boys), Al-Dawadmi Campus, Shaqra University, Riyadh, Saudi Arabia; ^5^ Department of Pharmaceutics, College of Pharmacy, Najran University, Najran, Saudi Arabia; ^6^ Department of Pharmaceutics, Faculty of Pharmacy, King Abdulaziz University, Jeddah, Saudi Arabia; ^7^ Department of Pharmacology and Toxicology, National Institute of Pharmaceutical Education and Research (NIPER)—Guwahati, Guwahati, India

**Keywords:** Parkinson’s disease, levodopa, levodopa-induced dyskinesia, pathophysiology, bradykinesia

## Abstract

Parkinson’s disease (PD) is the second leading neurodegenerative disease that is characterized by severe locomotor abnormalities. Levodopa (L-DOPA) treatment has been considered a mainstay for the management of PD; however, its prolonged treatment is often associated with abnormal involuntary movements and results in L-DOPA-induced dyskinesia (LID). Although LID is encountered after chronic administration of L-DOPA, the appearance of dyskinesia after weeks or months of the L-DOPA treatment has complicated our understanding of its pathogenesis. Pathophysiology of LID is mainly associated with alteration of direct and indirect pathways of the cortico-basal ganglia-thalamic loop, which regulates normal fine motor movements. Hypersensitivity of dopamine receptors has been involved in the development of LID; moreover, these symptoms are worsened by concurrent non-dopaminergic innervations including glutamatergic, serotonergic, and peptidergic neurotransmission. The present study is focused on discussing the recent updates in molecular mechanisms and therapeutic approaches for the effective management of LID in PD patients.

## 1 Introduction

Parkinson’s disease (PD) is a life-threatening progressive neurodegenerative disease, which is characterized by severe locomotor impairments including bradykinesia, tremor, and rigidity (Kalia and Lang, 2015; Mishra et al., 2021; Angelopoulou et al., 2021; Ahmad et al., 2022; Kaur et al., 2022). These symptoms are the manifestation of substantia nigral dopaminergic neuron loss (Kalia and Lang, 2015). Other abnormalities associated with PD are cognitive defects, psychiatric abnormalities, and neurodegenerative implications of levodopa-induced dyskinesia (LID) (Schapira et al., 2017). Levodopa (L-DOPA) is highly efficient and is the medication used for the mitigation of PD, but its prolonged use gives rise to motor abnormalities including dyskinesia. Dyskinesia may be mild at first but may develop into a debilitating symptom and may affect the quality of life of patients. More than 50% of patients with PD develop LID after 5 years of continuous treatment with L-DOPA, which worsens the quality of life in these patients (Fabbrini et al., 2007; Wu et al., 2019; Maan et al., 2020; Mandal et al., 2020).

Various types of movement disorders have been observed in LID which majorly include dystonia, myoclonus, chorea, or their combination. Generally, dyskinesia appears in the neck, jaw, tongue, face, waist, shoulder, trunk, and leg (Li et al., 2020). There are three types of dyskinesias identified to date, off-period dystonia, peak dose dyskinesia, and diphasic dyskinesia (Fabbrini and Guerra, 2021). Off-time dyskinesia is usually encountered during the night or early morning. Thus, administration of L-DOPA formulations having a longer half-life at night can be an effective way to treat off-time dyskinesia (Vijayakumar and Jankovic, 2016). However, diphasic dyskinesia is treated by increasing the dose of dopaminergic drugs administered; contrarily, treatment of peak dose dyskinesia is generally performed by reducing the dose of dopaminergic drug administered. However, in some cases, reduction of L-DOPA treatment leads to worsening of motor side effects (Fox et al., 2018). The management of LID remains a matter of grave concern as the diagnosis of the type of dyskinesia is a challenging issue. Furthermore, different types of dyskinesias can respond differently to a particular treatment.

LID is currently thought to be related to pre-/postsynaptic changes resulting in dopaminergic imbalance. Dyskinesia is associated with a series of events that include potent stimulation of dopamine (DA) receptors, low-protein and genetic mutations, and abnormalities in non-DA transmission (Bezard and Brotchie, 2001). Therefore, animal models are designed to explore the mechanisms involved in LID and to identify new pathological targets for drugs. Current mice and primate disease models are created by inducing PD by using MPTP or 6-OHDA toxins and then inducing LID by administering a daily dosage of L-DOPA (Bezard and Brotchie, 2001; Brotchie et al., 2007). In this article, we have discussed the pathophysiology, treatment trends of LID, and various preclinical and clinical evidence regarding this.

## 2 Basic Pathophysiology of L-DOPA-Induced Dyskinesia

The mechanism of LID is not clearly understood. However, continuous research has found that continued damage of nigral dopaminergic neurons produces deformities in the connection of the motor cortex and the striatum and makes a functional disturbance in basal ganglia, which can lead to the generation of involuntary abnormal movements, that is, LID (Bezard and Brotchie, 2001). The quantity and period of drug exposure play a crucial role in the development of dyskinesia. Along with that, the severity of LID also depends on the extent of neurodegeneration. PD patients and MPTP-treated primates having a higher degree of dopaminergic neuron degeneration show dyskinetic symptoms after the administration of L-DOPA. In general, rising and falling of the plasma L-DOPA level is the main reason for dyskinesia and motor defect (Thanvi et al., 2007).

**FIGURE 1:**
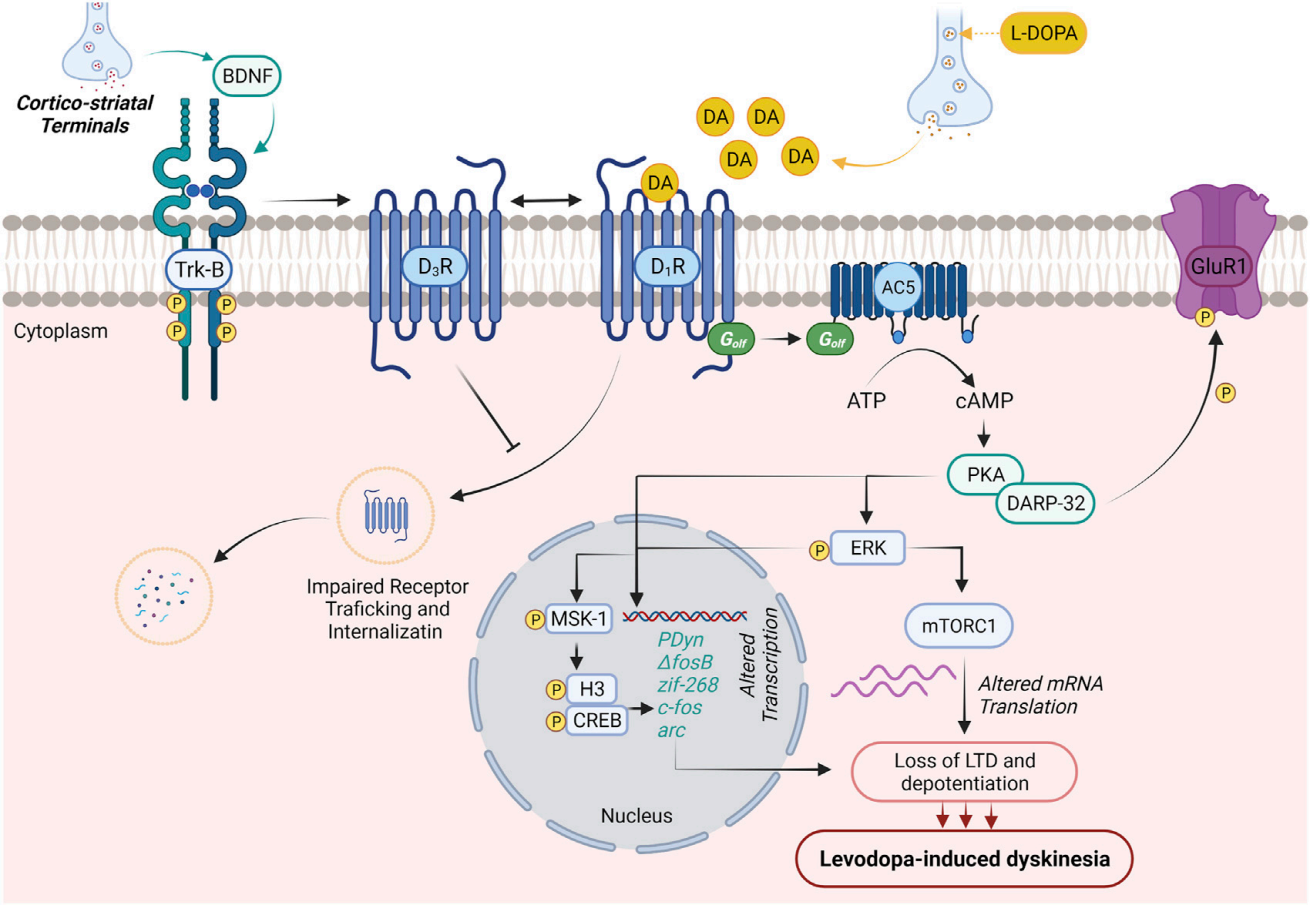
Molecular neurobiology of levodopa-induced dyskinesia. DA production generally regulates voluntary movements. In PD patients, initial dopaminergic neurodegeneration in substantia nigra pars compacta is asymptomatic. Loss of striatal–nigral neuronal sensitizes the D1 receptor on the medium spiny neurons of the direct pathway. This results in initial motor symptoms of PD (dyskinesia). The treatment with L-DOPA improves the initial motor symptoms and promotes BDNF release from corticostriatal neurons. The expressed BDNF potentiates the expression of D3Rs through the activation of Trκ-B receptors in nigrostriatal medium spiny neurons. Enhanced expression of D3Rs suppresses internalization and abnormal trafficking of membrane-bound D1Rs, thus intensifying D1R sensitization and associated dyskinesia. Activation of D1R by DA (released through L-DOPA) results in the activation of D1R/Gαolf/adenylyl cyclase 5 (AC5) machinery in nigrostriatal medium spiny neurons and causes cAMP-mediated hyperexpression of protein kinase A (PKA) and DARPP-32. The abnormal PKA/DARPP-32 expression results in hyper-phosphorylation of GluR1 and promotes the excitability of medium spiny neurons contributing to the loss of long-term depression and depotentiation and development of LID. On the other hand, activation of D1R results in the Ras/Raf/MEK/ERK signaling pathway, which further potentiates Ras/Raf machinery and regulates various transcription and translation processes regulating LID. PKA/DARPP-32 and/or ERK/Elk/MSK1 signaling migrates to the nucleus, leads to phosphorylation of CREB/histone H3, and augments the expression of immediate early genes (prodynorphin and ΔFosB), which are reported to contribute to the development of LID. Activated ERK further elevates mTORC1 expression-mediated mRNA translation and worsens LID.

Along with the advancement of the disease, the equal dosage of L-DOPA, which is generally required for alleviation of PD symptoms, causes dyskinesia. The main cause for this altered response pattern is not clear; however, the literature suggests that disturbance in between pre- and postsynaptic nigrostriatal DA transmission leads to motor complications. A schematic diagram describing major signaling abnormalities associated with LID in PD patients has been illustrated in Figure 1.

### 2.1 Improper Dopamine Storage, Neurotransmission, and Postsynaptic Alteration

Exogenously administered L-DOPA, a dopamine precursor, is converted to DA and stored in the vesicles of presynaptic dopaminergic neurons, which upon receiving stimuli releases it. This conversion and storage also take place inside the serotonergic nerve terminals. Even most of the exogenously administered L-DOPA is taken up by the serotonergic neurons and converted to DA. Dopamine transporter (DAT) transports this converted DA into the synaptic vesicles, where it is stored and released in a physiologically regulated manner (Corsi et al., 2021). Mainly, D_2_ autoreceptors and DAT buffer the release of DA into the synaptic cleft (Navailles and De Deurwaerdère et al., 2012). However, serotonergic neurons lack these mechanisms; thus, the physiological fine-tuning of DA release is absent in them. With the progression of disease neurodegeneration, the number of dopaminergic terminals which are required to modulate the production and controlled release of DA decreases. With the progression of time, dopaminergic neurons start to degenerate and serotonergic neurons become more relevant, and eventually, most of the striatal DA gets released, as false transmitters, from the serotonergic nerve terminals (Corsi et al., 2021). At an advanced stage of PD, a vast portion of dopaminergic neurons are lost, and the serotonergic terminal is mainly providing the DA release and conversion, which are devoid of the molecular mechanism for DA release management and feedback control. As a result, the DA concentration cannot be regulated, which leads to an abnormal swing in extracellular DA concentration following oral administration of L-DOPA (Mosharov et al., 2015; Fabbrini and Guerra, 2021). Fluctuations in the dopamine release patterns from the presynaptic neuron terminals give rise to abnormal responses, which were then carried out by postsynaptic neurons. These abnormal responses then give rise to fluctuations in motor coordination.

An important pathophysiological factor for LID is the shorter half-life after oral administration of L-DOPA, which produces a non-physiological pulsatile dopaminergic stimulus in the stratum region of the brain. This condition makes the intermediate level of DA dependent on the pharmacokinetics of external L-DOPA. Thus, the improvement from PD symptoms gets dependent on the administration of the L-DOPA dose. All these abnormalities ultimately give rise to progressive loss of the dopaminergic nerves (Bastide et al., 2015; Leta et al., 2019; Chen et al., 2020). Gradually, degeneration of presynaptic nigral neurons results in loss of DA in peak dose dyskinesia. After a few years of treatment, a new kind of dyskinesia starts to occur, which is known as wearing off dyskinesia. In this condition, worsening of PD symptoms takes place when the next dose of L-DOPA is due, and it improves after taking the new dose (Bezard and Brotchie, 2001; Bézard et al., 2003).

L-DOPA-induced aberrant dyskinetic activity could be due to deleterious plastic neurological implications at the postsynaptic levels. Between several different ideas supporting the role of postsynaptic variables in LID, one theory proposes that L-DOPA causes a stimulatory priming effect in Parkinson’s disease sufferers (Nadjar et al., 2009). Chronic introductions to medications that function as direct or indirect stimulants of cerebral DAT can cause sensitization to their cognitive stimulating characteristics, resulting in priming (behavioral sensitization). As a result, priming might occur in both drug usage and LID (Calabresi et al., 2008). Apparently, two required prerequisites for the induction and maintenance of LID that are thought to be a result of priming: high L-DOPA dosages and extensive DA denervation. L-DOPA generates dyskinesia with a constant spectrum of severity in dyskinetic rats, when given an appropriate dose, and results in severe striatal DA depletion (Calabresi et al., 2010).

Chronic drug therapy appears to cause unfavorable plastic alterations, which mostly occur in the postsynaptic region. After systemic administration of L-DOPA, this syndrome causes an excessively significant rise in extracellular DA (Abercrombie et al., 1990), which is coupled with defective monoamine oxidase-mediated DA breakdown, which further increases extracellular striatal DA concentration (Wachtel and Abercrombie, 1994). These huge changes in extracellular DA concentrations could then provoke abnormal postsynaptic responses. The increasing degradation of nigrostriatal dopaminergic neurons, which produces a reduction in DA holding space, is responsible for the difference between the severity of different disease phases. As a result, changes in L-DOPA fractions in the brain are solely dependent on the drug dosing period (Metman et al., 2000). Moreover, relative to stable responses, PET scans revealed fast oscillations in brain DA levels in individuals with PD and LID. Even though the drug-dosing cycle plays an important part in the vulnerability toward dyskinesias, other peripheral (gastric emptying and ingestion) and central (changes in precursor drug buildup in serotonergic neurons) elements are likely to play a part in the illness (Calabresi et al., 2010).

One further area which has lately been examined in mice persistently treated with L-DOPA, following unilateral 6-hydroxydopamine-induced lesion, is the anatomical location for the manifestation of LID (Buck et al., 2010). Dyskinesias were induced by locally injecting L-DOPA into the lesioned striatum, and on the other hand, regional L-DOPA administration of a similar dose in the ipsilateral globus pallidus and substantia nigra pars reticulata did not elicit dyskinesias, implying that the striatum is a particular target region for the activation of previously existent dyskinesias (Calabresi et al., 2010).

### 2.2 Altered Dopaminergic Pathways and Receptors

Three signaling pathways, PKA/DARPP-32, ERK, and mTORC1, were identified, which exert significant involvement in the pathophysiology of LID. All these pathways are interrelated and are activated by a common intercellular cascade triggered in nigrostriatal SPNs expressing D1 receptors. Chronic L-DOPA administration activates all of them, which alters the striatal synaptic plasticity. DARPP-32 signaling was observed to regulate ERK and mTORC1 pathways (Santini et al., 2012; Torkaman-Boutorabi et al., 2012).

Recent studies have demonstrated that D1 receptor activation leads to Shp-2 activation, which leads to activation of ERK1/2 and mTOR phosphorylation. This, in turn, induces the LID expression. This effect can easily be reversed by using a D1 receptor antagonist, which downregulates the end product of the D1R/Shp-2 pathway, p-mTOR, and p-ERK1/2 (Calabrese et al., 2020). Rapamycin was observed to be selectively affecting the mTORC1 pathway, without affecting DARPP-32 signaling, ERK pathway, and AMPA, NMDA expression (Santini et al., 2012).

After the onset of LID, the potential of D1 receptors to get associated with PKA and ERK1/2 increases. However, it was observed that after 2 weeks of L-DOPA treatment, D1 receptor-associated activation of PKA and ERK1/2 got blocked, due to oversensitivity of dSPNs (Wu et al., 2018). The D1R-dependent ERK1/2 phosphorylation was observed to be modulated by mGluR5, which modulates the mGluR5/PLC/PKC pathway without influencing PKA activity (Jones-Tabah et al., 2020). D1 receptor activation leads to activation of the D1R/cAMP/PKA pathway, which upregulates the expression of the Gα_olf_ protein. Gα_olf_ is a major stimulating G-protein, which upon activation upregulates the expression of cAMPs (Fieblinger et al., 2014). It was observed that chronic administration of L-DOPA increases the expression of Gα_olf_ in striatonigral SPNs and decreases its expression in striatopallidal SPNs (Alcacer et al., 2012). Knocking out casein kinase 2 (CK2) protein was also found to be linked with reduced Gα_olf_ protein expression in striatonigral SPNs. This also reduced the severity of LID. However, knockdown of CK2 in striatopallidal SPNs leads to increased severity of LID, which can be easily reversed by co-administration of caffeine and L-DOPA (Goto, 2017).

Upregulated Nurr1 transcription factor was found to be associated with increased cortically evoked firing rate and increased spine density of SPNs. Leucine-rich repeat kinase 2 (LRRK2) was observed to be influencing D1/D2 receptor-activated pathways. Interaction between them is not well understood, but this can help researchers to control LID in a better way (Sellnow et al., 2020). An anchoring protein, PSD95, dictates the distribution of dopaminergic receptors in the brain. An increased expression was noticed in DA depleted brain, which lowers the diffusion of D1R in the brain (Zhang J. et al., 2014; Divito et al., 2015). The expression of parkin protein also gets impaired, which leads to abnormal involuntary movements (Porras et al., 2012). Ca2+/calmodulin-dependent protein kinase IIα (CaMKIIα) binds to the same domain where the G_αi_ domain is located on the D2 receptor. This also indicates CaMKIIα D2 receptor activity *via* the adenyl cyclase signaling pathway. In the case of LID, increased interaction between CaMKIIα and D2 receptors was observed, and reduction in CaMKIIα concentration reduces LID, just like a D2 receptor agonist (Lyons et al., 2013).

An increased expression of D3 receptors was reported in animals with severe LID. An associated increase in the expression of GABA release was also noticed. D3 receptors can also perform the D1 receptor modulation action *via* affecting the ERK pathway. Upon deletion of the D3 receptor, levels of FosB, ERK, and H3 activities decreased along with the alleviation of LID symptoms, with no effect on L-DOPA treatment (Zhang S. et al., 2014; Albarrán-Bravo et al., 2019). A lack of D5 receptors is also associated with increased LID severity and decreased response to the L-DOPA symptoms (Payer et al., 2016).

### 2.3 Glutamatergic Hypothesis

In the past two decades, other than the dopaminergic system, numerous, non-dopaminergic systems are also observed to be involved in the pathophysiology of LID. First, significantly increased glutamatergic neurotransmission was observed in basal ganglia and the thermo-cortical circuit. In glutamate, ionotropic and metabotropic glutamate receptors are mainly involved in the excitatory effect. An excessive amount of AMPA and NMDA receptors were observed to be situated at the striatum of the patients and animals suffering from LID (Sgambato-Faure and Cenci, 2012; Guerra et al., 2019). Moreover, a histological study has shown that Glu (related to NMDA) is elevated in the striatum of the dyskinetic patients, but it has not shown any presence in non-dyskinetic patients. The sentence should be corrected as following: Abnormal glutamate release has been linked to development of LID in PD patients (Ahmed et al., 2011). Confirmation of the link between the elevated level of Glu and LID is also coming from pharmacological studies by using NMDA receptor antagonist drugs. By this, it has been confirmed that NMDA antagonist drugs are decreasing dyskinesia in animals and humans (Jongsma et al., 2001; Morin and Di Paolo, 2014).

It was also observed that metabotropic glutamate receptors (mGluR) are involved in the pathophysiology of LID. Additionally, Glu intracellular signaling is modulated by the MGlu receptor without affecting Glu excitatory action on synaptic neurotransmission. In the GPCR (G-protein coupling receptor), normally, three groups of mGlu are present, and they differ in ligand binding profile and sequence homology (Sebastianutto and Cenci, 2018). In the group 1 receptors (MGluR1, MGluR5) interact with phospholipase C beta and control intracellular calcium release by the inositol triphosphate formation along with protein kinase C activation. Both the group 2 (MGluR2, MGluR3) and group 3 receptors (MGluR4, MGluR6, MGluR7, and MGluR8) interact with the inhibitory G-protein and inhibit the formation of cAMP (Rondard and Pin, 2015; Sebastianutto and Cenci, 2018). Striatal PKA-related glutamatergic signaling and glutamatergic receptors (AMPA) play an important role in the expression and occurrence of LIDs. AMPA antagonists directly act on the glutamate receptors situated in M1 and are proven effective in alleviating LID. NMDA antagonists do not alleviate LID in rats when used as a monotherapy, but when co-administered with AMPA antagonists, they show LID-alleviating properties. This combination also potentiates the AMPA antagonists (Lindenbach et al., 2016).

### 2.4 Serotonergic Hypothesis

Serotonergic neurons convert 5-HT into DA, sort it, and then release it. This mechanism is referred to as the presynaptic serotonergic LID mechanism (Rylander et al., 2010; Sahin et al., 2014). As discussed earlier, serotonergic neurons play a critical role in the release of DA, and continuing this path, pharmacological lysis of serotonin raphe projections have shown to be blocking LID development in dyskinetic rats (Carta et al., 2007). As discussed earlier, in the advanced stages of PD, false dopaminergic neurotransmission is a serious pathological implication in the manifestation of LID symptoms. It was further established in hemiparkinsonian rats, where demolition of serotonergic innervation in the striatal region of the brain results in complete restoration of LID-like condition (Carta et al., 2007). The same effect was observed in non-dyskinetic rats upon removal of serotonergic afferents (Carta et al., 2007). Furthermore, an association of serotonergic innervations and serotonergic autoreceptors with the pathophysiology of LID was confirmed using other animal models also (Muñoz et al., 2008; Lindgren et al., 2010; Beaudoin Gobert et al., 2015). On the other hand, worsening of LID symptoms was observed after inducing the serotonergic innervations (exerts a tropic effect on serotonergic axons) in the striatum of parkinsonian rats, denoting that the striatum is the most important point of serotonergic implications (Tronci et al., 2017). Many articles support that irregular serotonergic movements are involved in the pathophysiology of LID. The involvement of the serotoninergic system in LID is supported by data showing significant striatal hyper-innervation in the parkinsonian animal (Gagnon et al., 2016). However, the serotonergic implication is not only restricted to the striatal region but also affects the parts wherever the serotonin innervation is present (Carta et al., 2018). Long-term L-DOPA administration was noticed to decrease basal serotonin release and metabolism, with associated downregulation of serotonin tissue content in the brain (Navailles et al., 2011). Thus, prolonged L-DOPA-induced reduction in 5-HT synaptic functionality may be implicated not only in the development of LIDs but also in the onset of non-motor health consequences of L-DOPA medication, such as anxiousness or melancholy, in which the serotonergic system plays a role (Navailles et al., 2011).

Radiological analysis has revealed an upregulation of serotonin transporter (SERT) expression in LID-affected patients (Sahin et al., 2014). Chronic L-DOPA treatment causes brain neurotrophic factor (BNDF) overexpression, which is associated with axonal growth of serotonin neurons, and further activates cAMP/PKA and mTORC pathways (Gagnon et al., 2016). In dopamine-depleted brains of PD-affected monkeys, striata and areas of dopaminergic denervation were found to be denser in SERT positive axons (Inden et al., 2012). Various selective serotonin reuptake inhibitors (SSRIs), like citalopram, paroxetine, and fluoxetine, decrease LID when co-administered with L-DOPA, while no such effects were observed with DA agonist, apomorphine. Thus, uptake blockage of serotonin is the preferred mechanism of anti-dyskinetic action (Bishop et al., 2012; Miguelez et al., 2016).

5HT_1A_ and 5HT_2B_ receptors are also passively involved in the prognosis of LID. They exist as autoreceptors in the axons and cell bodies of the serotonergic nerves and as heteroreceptors on striatal, as well as cortical neurons. As heteroreceptors, they control the release of GABA and glutamate in the cortical and striatal regions. As discussed earlier, the release of glutamate has some serious implications in LID. Thus, the activation of the 5HT_1A_ receptor leads to a release of glutamate, which exacerbates the LID symptoms (Carta et al., 2018). The role of serotoninergic system in LID has been validated through the animal models of 5HT1A and 5HT2B receptor agonists (animals having normalized serotoninergic receptor expression) and they have attenuated the dyskinetic symptoms produced by the L-DOPA administration. For example, vilazodone is a serotoninergic reuptake inhibitor and partial 5HT_1A_ agonist class of drug, which decreases the developing effect of LID without any interruption in motor effects of L-DOPA in 6-hydroxyDA-lesioned hemiparkinsonian rats (Meadows et al., 2018).

## 3 Approaches for Management of Levodopa-Induced Dyskinesia: Preclinical Status

The management of LID is often complicated as its pathogenesis is poorly understood. Based on the preclinical evidence, surgical and pharmacological approaches for the management of LID have been elaborated.

### 3.1 Pharmacological Approaches

**FIGURE 2:**
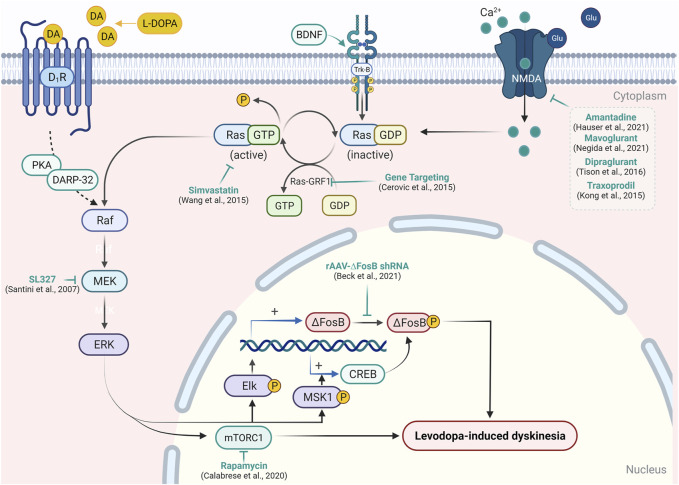
Targeting signaling upstream/downstream of Ras/Raf/MEK/ERK pathway modulation in LID. In the experimental model of PD, L-DOPA administration causes abnormal activation of the RAS/RAF/ERK pathway and results in the emergence of dyskinetic behavior. Activation of D1R, Trk-B, or NMDA receptor causes activation of RAS-GDP molecular switch by Ras-guanyl nucleotide-releasing factor 1 (Ras-GRF1), which facilitates the conversion of RAS-GTP to Ras-GDP. Ras-GDP further causes the activation of Raf protein kinase, which in turn leads to the phosphorylation of mitogen-activated protein kinase/ERK kinase (MEK) and ERK. Activation of ERK enhances expression of the transcription factor ΔFosB- and ERK-dependent activation of mTORC1, which results in the inhibition of long-term depression in striatal neurons and development of the abnormal involuntary movement in PD patients. Attenuation of Ras-GRF1 in the knock-out mice model results in the attenuation of LID. A similar reduction in LID was resulted in the inhibition of Ras (by simvastatin) and MEK (by SL327) expression. On the other hand, the inhibition of ΔFosB by short hairpin RNA and blockade of mTORC1 by rapamycin has been shown to mitigate LID. Various NMDA receptor antagonists have also been reported to reduce the expression of the Ras/Raf pathway and support their efficacy in LID.

For so many years, L-DOPA, which is a prodrug, is very effective in the treatment of parkinsonian motor symptoms. Chronic use of L-DOPA leads to several complications in PD patients. Response fluctuations and dyskinesia are examples of these complications. As a result, the management of the use of L-DOPA for treatment becomes a very important aspect. Many approaches participate that decide the best use of L-DOPA for the treatment of PD. Chemical approaches include two types of therapy, that is, dopaminergic therapy and non-dopaminergic therapy (Hauser et al., 2006; Encarnacion and Hauser, 2008). Recent updates on therapeutic interventions for LID (Santini et al., 2007; Kong et al., 2015; Wang et al., 2015; Calabrese et al., 2020; Beck et al., 2021) have been summarized in Supplementary Table S1 and illustrated in Figure 2.

#### 3.1.1 Improving Dopaminergic Therapy

LID is motor complications arising from pulsatile DA concentration in the brain. Theoretically, reaching a steady state of DA expression can precipitate dyskinesia. Thus, researchers have focused on developing therapies that provide continuous dopaminergic stimulation (CDS) to the brain. By doing this, the antiparkinsonian effects will remain unchanged, but it will be achieved without encountering dyskinesias.

Steady administration of L-DOPA abridged the risk of LID (Bibbiani et al., 2005). Many experimental studies showed that Parkinson’s patients are more prone to dyskinesia with L-DOPA than DA agonist monotherapy. The reason can be that DA agonists have more affinity for D2 receptors than D1 receptors (Sharma et al., 2015). Therefore, to manage dyskinesia in PD patients, the first step is to lessen the dose of L-DOPA during dopaminergic therapy given to the patient, but in some cases, it will worsen parkinsonism. Apart from this, we can also give repeated small doses of L-DOPA (Mazzucchi et al., 2015). During the management of dyskinesia, the focus is always to prevent the progress of dyskinesia and reduce the severity of dyskinesia. Instead of an intermediate release, the controlled release formulations of L-DOPA are helpful in delaying dyskinesia (Lee, 2001). Along with L-DOPA, dopaminomimetics having a longer half-life are administered to decrease pulsatile stimulation of DA receptors. In selected cases, COMT inhibitors are included as an example amantadine, which is recently given adjuvant to L-DOPA.

#### 3.1.2 Replacement With Other Dopaminergic Agents

As previously mentioned, prolonged formulation of DA agonists can be used in dyskinesia management because of their selective nature toward D2 receptors. D1 receptors are mainly responsible for emerging dyskinesia. Cabergoline, pramipexole, and ropinirole decreases the development of dyskinesia as compared to L-DOPA monotherapy during initial therapy in PD patients (Binde et al., 2020). DA agonists promote more continuous stimulation and restrict postsynaptic changes, which play the main role in L-DOPA inducing dyskinesia (Jenner, 2004). Pergolide, which is another example of a DA receptor agonist, delays the motor complication more than L-DOPA therapy (Bychkov et al., 2007). Bromocriptine monotherapy also lessens motor complication (Hussain et al., 2018).

Pramipexole, a D3 agonist, balances D1 DA receptor supersensitivity-associated LID. Early use of pramipexole not only prevents dyskinesia but also helps to treat motor fluctuations. Ropinirole is another example of a DA receptor agonist. Studies have claimed that ropinirole significantly lowers dyskinesia as compared to L-DOPA. Prolonged release of ropinirole has the potency to delay dyskinesia more than the increased dose of L-DOPA in patients. Rotigotine, a D1 receptor agonist, can also be used as a replacement for L-DOPA to prevent dyskinesia, but more investigation is required (Hutny et al., 2021).

#### 3.1.3 Adjuvant Therapy With L-DOPA

Catechol-O-methyltransferase inhibitors, also called COMT inhibitors, increase the obtainability of L-DOPA in the brain. The progress of dyskinesia can be overcome in PD patients by maintaining the plasma concentration of drugs in the body (Rascol et al., 2015). Entacapone and tolcapone are the drugs that help to reduce fluctuation in the concentration of L-DOPA. The only disadvantage is that tolcapone leads to liver toxicity. This disadvantage results in the development of new drugs which are safer and have enhanced activity (Deane et al., 2004). Opicapone belongs to the third-generation COMT inhibitor, a drug having safer and enhanced activity. This drug increases the bioavailability of L-DOPA, which has been proved in many experimental studies. Dyskinesia, insomnia, and dizziness are the disadvantages of opicapone (Hutny et al., 2021).

MAO-B inhibitors like safinamide, which also act on the glutamatergic system, help to improve the bioavailability of L-DOPA and increase its availability in spiny neuron synaptic clefts (Grégoire et al., 2013). In another study, safinamide is modulating the glutamate expression in the striatal region. Due to this, it has depicted significant motor complication reduction properties, without inducing troublesome dyskinesia. This fact can support its clinical use as an add-on therapy in the management of LID (Gardoni et al., 2018). Safinamide increases the bioavailability of L-DOPA when used as an ad on therapy, which makes this combination more effective in reducing motor complications (Schaeffer et al., 2014).

From a modification point of view, some alterations in therapy are included. The first modification is related to dose: the dose selection should be in a way that it gives more benefit. One experimental study proved that in the initial stages of treatment, if we give a dose of L-DOPA more than 600 mg, it may increase the chances of dyskinesia (Lee, 2001). In a modification, the dose of DA receptor agonists may increase or the daily dose of L-DOPA may decrease. Treatment of diphasic dyskinesia is more complex. In treatment therapy of diphasic dyskinesia, the addition of DA agonists or drugs that prolong L-DOPA, that is, COMT inhibitors or monoamine oxidase B inhibitors, are administered. D3 receptor antagonist IRL790 can also be given to a patient for maintaining psychomotor stability (Schaeffer et al., 2014; Hutny et al., 2021).

#### 3.1.4 Formulation-Based Approaches for Improved L-DOPA Therapy

Numbers of new formulations have been discovered by drug repurposing methods to manage dyskinesia induced by L-DOPA. The working of the new drug should be in a way that it reduces L-DOPA-induced dyskinesia but should not have any effect on antiparkinsonian activity. Scientists have tried to know the mechanism involved in the occurrence and expression of symptoms in LID. Pharmacokinetics of L-DOPA play an important role in the treatment of LID. We can achieve constant plasma levels by improving the delivery of a drug into the body (Rascol et al., 2015). XP21279 is a L-DOPA prodrug, which is converted into L-DOPA by carboxylesterases. This prodrug has a stable plasma concentration and can be used to diminish LID (Savola et al., 2003). CVT-301, an example of inhaled L-DOPA formulation used in PD patients, has also proven its ability in the improvement of motor complications (Stocchi et al., 2018).

L-DOPA/benserazide (polylactic-glycosic acid) microspheres (LBM), a novel therapeutic approach, have shown to be effective as an antiparkinsonian therapy without inducing dyskinesias. The effect was shown in a dose-dependent manner. LBM was observed to be not activating the D1R/Shp-2/ERK1/2 pathway, which was found to be activated in the 6-hydroxy DA rat model of LID (Fiorentini et al., 2013; Wan et al., 2017). These results were like the previous results, where it was observed that LBM reduces the frequency of LID. In various animal models, it was found that PKA/DARPP-32 signaling activation increases tau protein phosphorylation, which leads to subsequent activation of SNPs and results in LID. Dose-dependent inhibition of PKA/DARPP-32 signaling, tau protein phosphorylation, and activation of SNPs were also examined in LBM-treated animals. Thus, in the case of LBM-treated animals, the amount of dyskinesia encountered was also less frequent (Xie et al., 2014). Chitosan-coated nanoliposomes (CCN) is another advanced way to improve DA delivery in the brain. In Parkinsonian rats treated with CCN, it was observed that the induction of LID is less than that of L-DOPA-treated animals. CCN was observed to be reducing the expression of ERK1/2, DARPP-32, and FosB/DFosB, which were found to be upregulated in LID (Cao et al., 2016).

#### 3.1.5 Non-Dopaminergic Approaches

Non-dopaminergic activity influences symptomatic development, neurodegenerative processes and, more importantly, the development of available therapeutic adverse effects including LID. It has identified defects in several non-dopaminergic aspects of the pathway inside the basal ganglia. During the past few years, researchers have started to identify improper signaling *via* glutamatergic, adrenergic, serotonergic (5HT), cannabinoid, and opioid pathways in both stimulation and activation of LID at the cellular level (Brotchie, 2005).

##### 3.1.5.1 Adenosine A_2A_ Receptor Antagonists

Adenosine receptors play a crucial role in the development of LID. Adenosine interacts closely with DA and regulates the excessive glutamate neurotransmission in PD as well as LID (Morin et al., 2016). Adenosine A_2A_ receptor antagonists were observed to be reducing excessive striato-pallidal and subthalamic neuronal activity (Blandini and Armentero, 2012). In a study, it was observed that MPTP-treated monkeys with dyskinesia have an upregulated expression of adenosine A_2A_ receptor, compared with non-dyskinetic MPTP treated monkeys (Morin et al., 2016). Hence, adenosine receptors have gained increased attention from researchers.

ST1535, an adenosine A2 receptor antagonist, was examined to be effective as an antiparkinsonian agent, and it significantly reduces the requirement of L-DOPA dose. Thus, using this as an add-on therapy along with L-DOPA may significantly delay the development of LID (Rose et al., 2006). Caffeine also shows adenosine A2 receptor antagonism action. It was examined that caffeine was able to alleviate LID symptoms in 6-OHDA-treated mice. The mechanism of action was found to be blockage of adenosine A_1_ or A_2A_ receptors (Xiao et al., 2011). Istradefylline, another adenosine A2 receptor antagonist, was observed to be effective in improving motor complications associated with PD without provoking dyskinesia. Therefore, it has the potential to be used as a monotherapy in PD; in that way, it will eliminate the occurrence of LID. However, more research is required to explore its potential (Uchida et al., 2015). Contrastingly, SCH 412348, an adenosine A2 receptor antagonist, does not alleviate LID when co-administered with L-DOPA in 6-OHDA-treated rats (Jones et al., 2013). Thus, using a combination therapy of L-DOPA and adenosine A2 receptor antagonists to treat LID might not carry that much potential. However, more research is needed to confirm it.

##### 3.1.5.2 Adrenergic Receptor Antagonists

The adrenergic system also plays a crucial role in the development of PD and LID. In a study, it was observed that the noradrenergic system modulates the LID severity. In that same study, it was also demonstrated that in 6-OHDA-treated hemiparkinsonian rats, the use of alpha- and beta-adrenergic receptor blockers can alleviate the development of LID (Barnum et al., 2012). Alpha-2 adrenergic receptor blockers idazoxan, rauwolscine, and yohimbine were also observed to be reducing LID in the MPTP-lesioned primate PD model. Idazoxan as monotherapy has no beneficial effect on PD or LID, but when it was used in combination with L-DOPA, it not only alleviated LID but also increased the t_max_ more than two times (Paredes-Rodriguez et al., 2020). Propranolol, a potent beta-adrenergic receptor blocker, was also observed to be reducing LID in hemiparkinsonian rats. The researchers found that LID-alleviating effects of propranolol were mediated *via* mitigation of L-DOPA-induced extra physiological efflux of DA (Bhide et al., 2015). Fipamezole (JP-1730) is also a potent alpha-2-adrenergic blocker. It was observed to be reducing LID in MPTP-lesioned primate PD models. The combination of fipamezole and L-DOPA increases the duration of action of L-DOPA by 66%, compared with L-DOPA alone (Savola et al., 2003). Thus, adrenergic receptor antagonists have great potential as an adjunct to classical L-DOPA therapy. It can be beneficial in increasing the duration of the therapy and providing continuous dopaminergic stimulation.

##### 3.1.5.3 Glutamatergic Antagonists

There are two types of glutamatergic receptors: First is ionotropic glutamatergic receptors (iGluRs) that mediate fast excitatory neurotransmission, and the second one is metabotropic glutamatergic receptors (mGluR) that mediate slow excitatory neurotransmission. iGluRs are classified into NMDA, AMPA, and kainite (KA) receptors (Morin and Di Paolo, 2014; Fabbrini and Guerra, 2021). Higher striatal expression of NMDA and AMPA receptors was reported previously in MPTP-treated dyskinetic monkeys (Morin and Di Paolo, 2014). However, NMDA and AMPA receptor blockage was observed to be alleviating the development of LID in 6-OHDA rats (Fabbrini and Guerra, 2021).

Mavoglurant is an antagonist for the glutamate mGluR5 receptor. Ionotropic and metabotropic glutamate receptor antagonists are subjected to examination for their possible involvement in the alleviation of LID since overactivity of glutamatergic pathways has been linked to the development of LID in animal models (Jimenez-Urbieta et al., 2015; Trenkwalder et al., 2016a; Negida et al., 2021). The striatum is rich in metabotropic mGlu5 receptors, which are related to NMDA receptors. mGlu5 receptor antagonists appeared to be beneficial in correcting aberrant movements in 6-hydroxy DA (6-OHDA)-lesioned macaques in preclinical trials. Human trials of mavoglurant, a metabotropic mGlu5 receptor antagonist, have had mixed results. In addition, the medication has been linked to several potentially dangerous side effects such as hallucinations and dizziness. MGluR4PAM in several animal models of PD, a positive allosteric modulator of mGluR4 (VU0364770), elicits significant antiparkinsonian effects, but it fails to affect the development and evolution of LID in unilaterally 6-OHDA-lesioned rats (Iderberg et al., 2015; Tison et al., 2016).

Traxoprodil is an NR2B subunit-specific NMDA receptor antagonist. It was observed that administration of traxoprodil revealed 30% reduction in LID severity, but there was no reduction in motor abnormalities or certain cognitive difficulties associated with therapy (Nutt et al., 2008; Kong et al., 2015). Memantine functions as a non-competitive NMDA receptor antagonist. In an experiment with 6-ODHA-lesioned rats, both memantine and amantadine greatly decreased LID, but the alleviating effect of memantine was found to be, in a few days, showing a rapid tolerance to the anti-dyskinetic action of this medicine (Jankovic and Clarence-Smith, 2011). In contrast, riluzole, an NMDA receptor antagonist, did not elicit any anti-dyskinetic effect (Bara-Jimenez et al., 2006). CI-1041, another NMDA receptor antagonist, was observed to be inhibiting the development of LID in parkinsonian monkeys. The mechanism of action observed was the downregulation of the striatal mGlu5 receptor (Hadj et al., 2004).

##### 3.1.5.4 Serotonergic Agonists

Chronic L-DOPA therapy and decreased DA levels in the brain lead to alterations in the brain’s serotonergic system. It was observed that modification of the serotonergic system exerts an inhibitory effect on LID. However, careful administration is required as an overdose of serotonergic agonists can lead to symptomatology (5-HT syndrome), which shows PD-like symptoms (Hernandez et al., 2019). Buspirone, a partial 5-HT1A receptor agonist, was examined to be preventing the development of LID in rats without altering the STN electrical activity. An observed mechanism was found to be the regulation of GABA and Glu release, along with burst activity in pars reticular of SN (SNr) (Vegas-Suárez et al., 2020).

8-Hydroxy-2-dipropylaminotetralin (8-OH-DPAT), a 5-HT1A receptor agonist, was reported to be reducing LID development *via* downregulating D1 and D2 agonism-induced cortical gamma overactivity. However, associated frequent 5-HT syndrome development limits its further application (Nahimi et al., 2012; Dupre et al., 2016). SSRIs were also evaluated for this purpose. Citalopram, an SSSRI, was observed to be alleviating LID development without inducing 5-HT syndrome (Lindenbach et al., 2015). Another 5-HT_1A_ receptor agonist, BMY-14802, was also evaluated to be showing LID reducing effect in a dose-dependent manner. It not only reduces L-DOPA-induced dyskinesias but reduction of DA agonism-induced dyskinesias was also reported. The chances of developing 5-HT syndrome after treatment were also very low as its activity can be easily reversed by using a 5-HT1A receptor antagonist (Bhide et al., 2013). 5-HT_3_ receptor antagonism is another possible checkpoint in the treatment of LID. Ondansetron, a well-known 5-HT_3_ receptor antagonist, was examined for this purpose. After 23 days of continuous co-administration with L-DOPA, a significant reduction in LID development was noticed between Ondansetron + L-DOPA-treated group and only L-DOPA-treated groups. These results suggest that 5-HT_3_ receptor antagonism carries potential in controlling the development of LID, but more research is needed to explore its maximum potential (Aboulghasemi et al., 2018).

Serotonin precursor, 5-hydroxy-tryptophan (5-HTP), was also examined to bear anti-dyskinetic properties. The basic mechanism of its activity is the upregulation of cytoplasmic serotonin, which competes with DA in serotonergic neurons and decreases the DA release from nerve terminals (Maffei, 2020). 5-HTP was reported to be alleviating LID in 6-OHDA-treated PD mice. When co-administered with L-DOPA, it neither loses its LID-alleviating properties nor decreases the antiparkinsonian effect of L-DOPA (Tronci et al., 2013). Thus, it can serve as a potent adjunct to L-DOPA therapy, which can minimize the development of LID.

In rat models of LID, levetiracetam has been found to decrease abnormal involuntary movements in a dose-dependent manner. While the mechanism for this improvement is unknown, it is thought to occur as a result of its effect at many locations in the LID cascade, including a change in the expression of specific transcription factors and phosphorylated kinases in the striatum (Du et al., 2015). In an MPTP non-human monkey model of PD, levetiracetam was observed to enhance the effects of amantadine (Hill et al., 2004). Levetiracetam has been proven to be effective in treating LID in several open-label clinical studies (Tousi and Subramanian, 2005).

##### 3.1.5.5 Opioid Drugs

The idea that increased signaling by opioid peptides may promote to the development of LID has piqued interest for more than a decade because the opioids affect the neurotransmission in the basal ganglia, and significant alteration in opioid signaling is generally encountered in PD patients (Johansson et al., 2001). Thus, drugs modulating this system can prove to be an effective alternative therapy to the standard anti-dyskinetic therapy.

Combination treatment of L-DOPA and nalbuphine (κ subtype agonist and µ subtype antagonist) was examined to be downregulating the expression of various LID markers, like ∆FosB, prodynorphin, dynorphin A, Cdk5, and Thr34 phosphorylation of DARPP-32, to normalized levels. No adverse events were encountered during this study. The sedative effect of nalbuphine was also not induced by L-DOPA (Potts et al., 2015). To find out the potential of µ receptor antagonism monotherapy in alleviating LID, another study was performed on the 6-OHDA PD rat model. In this experiment, it was found that selective µ receptor antagonism is not able to improve dyskinesias in rats (Bartlett et al., 2020b). Thus, the results obtained by using the L-DOPA and nalbuphine combination therapy might be an outcome of the therapy’s dualistic nature. However, µ receptor agonism monotherapy was able to downgrade the incidence of LID in the MPTP parkinsonian rat model (Hutny et al., 2021).

Mu-delta opioid receptor agonism can also be beneficial in the treatment of LID. Lactomorphin, a mu-delta opioid receptor agonist, was examined to exert antiparkinsonian activity along with reduced incidence of dyskinesia in 6-OHDA-treated male SD rats. It reduced the AIM score significantly when compared with the untreated animals (Flores et al., 2018). Cyprodime, ADC-02520849, and ADC-02265510 are three opioid receptor agonists. They were examined to be alleviating LID symptoms along with reduction of LID severity scores in MPTP-treated primate models of PD (Bezard et al., 2020). Thus, the use of opioid drugs is an effective pharmacotherapeutic way to reduce dyskinesias associated with L-DOPA treatment. However, much research is needed to evaluate such possibilities.

##### 3.1.5.6 Nitric Oxide Modulators

Loss of dopaminergic neurons in the substantia nigra of the brain is the main characteristic feature of PD. However, along with DA depletion hindrance in nitric oxide (NO), neurotransmission was also observed to be hindered during PD. DA by direct acting on DA receptors situated in the striatal nNOS interneurons facilitates NO production. In PD, there is a significant decrease in the expression of nNOS-containing neurons and nNOS mRNAs. NO is proven to play a crucial role in the control of motor functions (Pierucci et al., 2011; Lorenc-Koci et al., 2017). Thus, it can be concluded that NO hypofunction can play a crucial role in the development of PD and LID.

In a study, it was observed that when NO donor molsidomine was co-administered with L-DOPA, the expression of DA gets increased, which in turn decreases the incidence of LID (Lorenc-Koci et al., 2013). The anti-dyskinetic property of molsidomine was also demonstrated in genetically susceptible PD mice (Solís et al., 2015). Upregulation of NO-soluble guanylyl cyclase–cGMP signaling also plays a vital role in LID. Methylene blue, a potent inhibitor of it, can alleviate LID, when co-administered with L-DOPA or when injected into the lateral ventricle of the brain as a monotherapy (Pierucci et al., 2011; Bariotto-Dos-Santos et al., 2019). Thus, the use of NO modulators carries the potential to be included in the management of LID. However, clinical evidence regarding this is still missing in the literature. More research is required to reveal its true potential in the management of LID.

## 4 Clinical Management of Levodopa-Induced Dyskinesia

### 4.1 Surgical Approaches

Neurosurgical approaches have shown to be the most effective in providing sustained relief from LID in PD patients. Mainly, neurosurgical approaches associated with basal ganglia and cerebral cortex have shown to be the most effective in treating LID.

#### 4.1.1 Deep Brain Stimulation

DBS is a highly effective and the most preferred procedure in LID patients having advanced PD, drug refractoriness, and motor complications associated with L-DOPA therapy (Fasano et al., 2012; Munhoz et al., 2014). Various randomized control trials (RCTs) have proven that DBS reduces the PD symptoms, dyskinesias, and reduces the need for dopaminergic stimulation in LID (Kopell et al., 2006; Nazzaro et al., 2013). There are mainly two widely persuaded targets in the human brain that are being stimulated by DBS, that is, subthalamic nucleus (STN) and globus pallidus internus (GPi). Many RCTs have proven STN DBS and GPi DBS to be more successful in alleviating LID symptoms than medical treatment (Deuschl et al., 2006; Weaver et al., 2009). Few studies have demonstrated that STN DBS is more effective at reducing the required medication dose in LID, and these studies have also confirmed that STN DBS application does not lead to worse neuropsychiatric and cognitive outcomes than those of GPi DBS (Odekerken et al., 2016; Xie et al., 2016; Elgebaly et al., 2018). There are some conflicting pieces of evidence, whether the beneficial mechanism of DBS in LID is due to its direct stimulation, reduction of L-DOPA dosage, or a blend of both. However, some studies suggested that the effects are target-dependent. Like in the case of GPi DBS, the dyskinesia scores (UPDRS IV) get improved without reducing the required L-DOPA dose (Volkmann et al., 2004; Follett et al., 2010; Moro et al., 2010; Odekerken et al., 2016; Bonenfant et al., 2017), whereas in the case of STN DBS, the mechanism is of mixed type (Castrioto et al., 2011; Zibetti et al., 2011; Munhoz et al., 2014; Krishnan et al., 2016).

Classical DBS is mainly based on frame-based and frameless stereotactic methods with microelectrode recording (MER, for electrode placement confirmation). Portable imaging techniques (O-arm) have also been used in some studies, with or without the use of MER, to improve the accuracy and safety associated with DBS target placement (Burchiel et al., 2013; Frizon et al., 2018). Another technique, namely, frameless iMRI-guided DBS, was successfully investigated to have significantly greater accuracy than that of the classical DBS. It uses interventional magnetic resonance imaging (iMRI), and the electrode placement in it is guided by a skull-mounted aiming device. It is performed in anesthetized patients with the assistance of a real-time MRI scanner. The real-time MRI can account for the brain shift after the skull is opened, contrary to the classical DBS, where stereotactic placement based on preoperative imaging is the only option (Nakajima et al., 2011). A second-generation iMRI-guided DBS with improved operator control and fully integrated software has also been reported to be used with even greater lead placement accuracy and favorable patient outcomes (Ostrem et al., 2016).

Recently, frame-based stereotactic DBS has also been used along with iMRI (Matias et al., 2018). The iMRI-guided DBS has certain advantages over classical DBS, like improved electrode placement accuracy, streamlined procedure (as the patients are anesthetized), improved patient compliance (as performed under general anesthesia), faster lead placement times, and incorporation of MER for target confirmation (Ostrem et al., 2013). The iMRI-guided DBS is a very costly procedure, along with the logistical challenges of maintaining detectable intraoperative MRI and scheduling the procedure time on a diagnostic scanner. So, the cost to benefit ratio for patients included in this procedure must be evaluated with great importance (Azmi et al., 2016).

Classical DBS needs calibration for every patient to avoid side effects like dyskinesia, as incorrect stimulation parameters can lead to dyskinesia. To address this drawback, an adaptive or closed-loop DBS has been developed. In adaptive DBS, a sensory electrode and a modulating electrode are applied on the motor cortex and basal ganglia region, respectively. They help in monitoring the brain activity patterns associated with dyskinesia. Upon detection of these patterns, a feedback loop gets activated, which decreases the stimulation delivered to the target region (Swann et al., 2018). An adaptive DBS study was successfully performed using a novel narrowband gamma oscillation as a biomarker for dyskinesia in the motor cortex to modulate the target stimulation delivered (Swann et al., 2018). They also reported 38–45% less energy consumption than classical DBS to maintain the same therapeutic efficacy (Dastin-van Rijn et al., 2021). Recently, more responsive adaptive DBS systems have been developed using accelerometer-based technology, which detects movements by utilizing peripheral kinematic sensors to detect dyskinesia (Rojas Cabrera et al., 2020).

#### 4.1.2 Surgical Ablation

Surgical ablation is another well-persuaded procedure in LID. Ablation of globus pallidus is preferred when several factors, like situations with fewer resources, difficulties in follow-up for several years, and risk of complications from electrode implantation, make DBS less practical to follow (Sharma et al., 2020). Unilateral pallidotomy has shown to result in long-lasting and sustained improvement of LID, tremor, rigidity, bradykinesia, gait, and balance. Contralateral and ipsilateral improvements in LID were also reported by following this procedure (Vitek et al., 2003; Upadhyay et al., 2015). However, it was reported that bilateral STN DBS produced greater improvements in motor and bradykinesia symptoms than unilateral pallidotomy (Esselink et al., 2004).

Ultrasound ablation or focused ultrasound ablation is a newly developed and minimally invasive procedure, which has been successfully examined to be highly effective in the treatment of LID (Magara et al., 2014; Na et al., 2015; Schlesinger et al., 2015; Zaaroor et al., 2018). It is a magnetic resonance-guided focused ultrasound (MRgFUS) technique, which has been successfully incorporated with pallidothalamic tractotomy (Fine et al., 2000) and unilateral pallidotomy (Na et al., 2015) procedures, and it resulted in better outcomes than conventional surgical ablation procedures. Focused ultrasound ablation showed to improve the UPDRS motor scores and contralateral dyskinesia scores in LID patients (Magara et al., 2014; Na et al., 2015; Schlesinger et al., 2015; Zaaroor et al., 2018). Thus, it has great potential in the management of LID.

#### 4.1.3 Transcranial Magnetic Stimulation

TMS is one newly implemented non-pharmacological method to treat LID. TMS is a non-surgical procedure, in which a specific area of cortex is stimulated by recurrent single pulse electromagnetic conduction that emerges from a coil, which is coupled to a pulse generator. It has been observed that the excitability in the cortical region gets increased after application of high-frequency stimulation, and it gets decreased by the application of low-frequency stimulation, but in the case of theta-burst stimulation (TBS), intermittent stimulation increases cortical excitability, whereas continuous stimulation reverses (Pascual-Leone et al., 1994; Chen et al., 1997; Huang et al., 2005).

A study suggests that LID is mainly a manifestation of primary disinhibition of motor cortex and secondary excessive outflow of pallidothalamocortical motor loop (during peak dosing) (Rascol et al., 1998). Overlapping of these quantifiable cortical changes with LID symptoms provides strong evidence toward the involvement of cortical dysfunction in the pathophysiology of LID (Rascol et al., 1998). Thus, applying stimulation to the cortex region to undo its dysfunctions is a rational LID treatment approach.

Although some studies have demonstrated that repetitive TMS (rTMS) with lower frequency (1 Hz) can produce a significant decrease of dyskinesia symptoms in patients, but the effect is not long term, recurrent rTMS treatment is needed to maintain the reduced dyskinesia state for a longer duration of time (Wagle-Shukla et al., 2007; Filipović et al., 2009). However, the long-term effects of rTMS are still debatable as there are study limitations like insufficient statistical power, protocol variation, short follow-up period, and lack of standardized control procedure. Due to these limitations, the dopaminergic effects of rTMS are not proven yet (Strafella et al., 2006; Elahi et al., 2009; Benninger et al., 2012).

#### 4.1.4 Implanted Motor Cortex Stimulation

MCS is a neurosurgical procedure that allows implantation in both epidural and subdural levels, and due to that, long-term, repetitive electromagnetic stimulation is delivered to the cortex. However, epidural implantations are preferred over subdural implantations because of no dampening of therapeutic stimulation by CSF and less serious complications in it (Bezard et al., 1999; Saitoh et al., 2003; Manola et al., 2005; Delavallee et al., 2008). MCS works on the same principles as TMS. The mechanism of action of the MCS procedure is uncertain, but some studies on primate PD models have proven that “electromagnetic stimulation dampens the abnormal oscillations taking place between the cortex and basal ganglia in PD, normalizes hyperactive structures in these areas, and reactivates hypoactive structures” (Drouot et al., 2004; Lefaucheur, 2009). Implanted MCS has shown promising results in reducing L-DOPA dose; hence, delaying the onset of LID (Canavero and Paolotti, 2000; Cilia et al., 2007). However, some studies have reported conflicting results. In those studies, it has been observed that a lower frequency of stimulation produced negative results, whereas higher frequency stimulation produced positive results (Lefaucheur, 2009). Despite these conflicting results, implanted MCS has great potential in the management of LID. Further research can fortify its application in LID with greater effectiveness.

### 4.2 Pharmacotherapeutic Approaches

Pathological knowledge of LID has increased vastly in the last decade. Many pharmacotherapeutic approaches have shown promising results in their preclinical studies, but their clinical results are not satisfactory. The present clinical status of LID management has been summarized in Supplementary Table S2.

There is profound evidence present in the literature regarding the treatment of LID. The main goal of LID treatment has been the reduction or prevention of LID or to reduce its onset, maintaining the excellent antiparkinsonian effect of L-DOPA (good on time). To achieve this goal, researchers have mainly focused to reduce the L-DOPA dose, providing continuous dopaminergic stimulation, and identifying non-dopaminergic targets to treat LID.

Continuous usage of lower doses of L-DOPA is the most preferred clinical strategy in the management of LID (Scott et al., 2016). There is a clear correlation between the dose of L-DOPA used and the severity and likelihood of the dyskinesia developed. It was observed that PD patients receiving higher doses of L-DOPA are more likely to develop LID (Fahn, 1999; Verschuur et al., 2019). Along with that, adjuvant therapy of DA agonists and MAO inhibitors was also evaluated in various seminal studies, with an aim of reducing the L-DOPA dose; thus, delaying the onset of LID (Holloway et al., 2005; Watts et al., 2010). However, no long-term benefit has been observed. A new extent release capsule of carbidopa and L-DOPA named IPX066 has also been discovered. This capsule contains immediate and sustained-release pellets, and has become superior over L-DOPA + carbidopa + entacapone in the treatment of PD (Hauser et al., 2013). L-DOPA carbidopa subcutaneous pumps are also used in PD patients. This subcutaneous pump helps reduce fluctuation in L-DOPA concentrations (Giladi et al., 2015).

Due to the failure of these approaches, the concept of continuous dopaminergic stimulation (CDS) has emerged. This technique makes use of longer acting dopaminergic agents, which provide more constant and long-lasting dopaminergic stimulation to the dopaminergic receptors. Rotigotine, apomorphine-like drugs, entacapone/L-DOPA, and L-DOPA carbidopa intestinal gel (LICG)-like formulation approaches have been taken to provide CDS, but they have proven beneficial only in advanced PD and not in LID. However, they are reported to be reducing the occurrence of motor complications and dyskinesia. Thus, using them along with other preventive medication can increase the quality of life of patients suffering from LID (Olanow et al., 2020).

DBS is the most effective technique by which CDS and subsequent alleviation from LID can be achieved. Stimulation of the subthalamic nucleus and internal globus pallidus has shown to be beneficial in LID as it induces the striatal DA release, which provides more sustained CDS (Fox et al., 2018). However, despite the benefits, cost, lack of accessibility, invasiveness, and side effect profile limit their use on human subjects (Buhmann et al., 2017).

MAO-B inhibition has also proven to be effective in the management of LID. Safinamide is a water-soluble orally given aminoamide that has two mechanisms of action: MAO-B inhibition and glutamate release inhibition. In a two-year period, in PD, a prospective double-blind placebo-controlled study is being conducted. Safinamide treatment was found to reduce the motor complications without worsening PD complications (Borgohain et al., 2014). When compared to placebo, long-term usage of safinamide did not increase the risk of dyskinesia, and 100 mg of safinamide per day reduced dyskinesia in patients who had more severe dyskinesia at baseline and was well accepted by patients (Borgohain et al., 2014).

Zonisamide works *via* increasing DA production, inhibiting MAO-B, inhibiting glutamate release, and inhibiting sodium and T-type calcium channels, among other things. Since studies indicated an improvement in PD symptoms with a very low frequency of side responses such as dyskinesia and hallucination, zonisamide has been approved in Japan for use as a supplementary therapy in PD patients. Zonisamide lowers off-time in PD patients with wearing off, according to a double-blind RCT including 422 patients (Murata et al., 2015). Zonisamide is effective in the treatment of PD motor symptoms, although it is still considered experimental for other indications, such as the therapy of LIDs (Seppi et al., 2011).

Along with dopaminergic alteration, scientists have identified many non-dopaminergic targets/add-on therapies also. Metabotropic glutamate receptor agonism is one the most promising aspect of it. Dipraglurant and mavoglurant (AFQ056), two selective metabotropic glutamate receptor agonists, have shown to be significantly reducing dyskinesia without worsening PD symptoms (Stocchi et al., 2013; Tison et al., 2016). Amongst them, dipraglurant has shown a significant reduction in peak dose dyskinesia, along with rapid absorption (T_max_ = 1 h) (Tison et al., 2016). This fact can support the rational use of dipraglurant in the management of acute peak dose dyskinesia cases, but its efficacy in such cases is yet to be evaluated. However, in another clinical study, mavoglurant (AFQ056) has failed to show LID-alleviating effects (Negida et al., 2021). Thus, the use of mavoglurant in the management of LID is questionable.

Out of several add-on therapies evaluated, amantadine, which is a non-selective NMDA receptor antagonist, is the most widely used and has proven to be the most effective add-on treatment in clinical studies (Fox et al., 2018; Hauser et al., 2021; He et al., 2021). Extended-release formulations of it were observed to be significantly increasing the on time, but it does not affect the dyskinesia (Hauser et al., 2021). However, due to side effects like confusion and hallucinations, their human application is limited (Hubsher et al., 2012). FDA has approved the use of a long-acting amantadine formulation for LID treatment in 2017. It provides a more stable plasma concentration and bioavailability than that of the immediate release forms, that is, supporting the concept of CDS (Müller and Möhr, 2019). In one phase III clinical study, it was observed that single daily dosing of amantadine significantly reduces LID in comparison to placebo-treated group (Pahwa et al., 2015; Paik et al., 2018). In another study of extended-release amantadine formulation, it was observed that a reduced level of LID was achieved, and that reduced expression was maintained for a median treatment period of 1.9 years (Tanner et al., 2020). However, its chronic clinical use for a longer period still needs to be evaluated. 100 mg of amantadine twice daily resulted in a significant decrease in total dyskinesia by 24%, and a significant reduction in duration of dyskinesia was also observed in a single double-blind placebo-controlled study involving 24 patients (Snow et al., 2000). Dyskinesias deteriorated within a week in patients who stopped using amantadine, according to a 3-month multicenter double-blind RCT comparing patients who were kept on amantadine vs. those who switched to a placebo. Apathy and tiredness were more common worsening conditions in these individuals (Friedman et al., 2014). Though the effects of amantadine anti-dyskinetic were thought to disappear after a few months, clinical investigation on 32 PD patients with LID, who received amantadine for more than a year, has shown improvement in dyskinesia. In contrast, some studies suggested that the anti-dyskinetic effect was not the same (Wolf et al., 2010). The findings revealed that amantadine remained efficient in treating dyskinesia longer than a year after therapy began, with no adverse signs recorded in patients. The LID symptoms were observed to deteriorate in the group which received a placebo treatment. Furthermore, chronic amantadine treatment was associated with the development of hallucinations, sedation, myoclonus, livedo reticularis, hair loss, and edema (Seppi et al., 2011; Vijverman and Fox, 2014), which suggests the careful monitoring of the patients.

Another most promising non-dopaminergic approach to treat LID is the 5-HT (5-HT_1A_ and 5-HT_1B_) receptor agonism. 5-HT_1A_ and 5-HT_1B_ receptor agonism have also gained research interest, as serotonergic stimulation is associated with DA release and activation of 5-HT_1A_, 5-HT_1B_ receptors, which can lead to increased serotonin release in the brain (Carta et al., 2007). Eltoprazine, a 5-HT_1A_, and a 5-HT_1B_ receptor agonist have been shown to be alleviating dyskinesia without interfering with the L-DOPA activity. It was also reported to be well tolerated amongst the patients (Svenningsson et al., 2015). Thus, this can be a possible add-on therapy to L-DOPA in controlling LID. In a randomized placebo-controlled double-blind pilot trial of nine PD patients, on time without dyskinesia or with non-troublesome dyskinesia increased significantly (Zesiewicz et al., 2005). Another multicenter double-blind placebo-controlled crossover experiment in 38 individuals with LID found that LID at dosages of 500 and 1,000 mg per day resulted in a substantial reduction in time (Stathis et al., 2011). However, a nine-patient open-label trial found that levetiracetam was not well tolerated in PD patients, with most patients experiencing sleepiness and increasing dyskinesia (Lyons and Pahwa, 2006).

In a phase 1 or 2 a dose-finding trial, eltoprazine, an agonist of presynaptic 5-HT_1A_ and 5-HT_1B_ receptors, established its anti-dyskinetic efficacy at 5 or 7.5 mg without affecting normal motor responses to L-DOPA (Svenningsson et al., 2015). Pardoprunox is a partial and full agonist at D_2_ and D_3_ receptors, as well as a low-affinity agonist at D4, α-2 adrenergic, and 5HT_7_ receptors. Because of its partial agonist action at DA receptors, this medication is thought to have a reduced risk of dyskinesia than other DA agonists. An RCT found a substantial increase in on time without bothersome dyskinesias when pardoprunox was titrated to as high as 42 mg per day (Hauser et al., 2009). A 12-week randomized placebo-controlled trial found that pardoprunox rose on time without causing troublesome dyskinesia, although the research had a high dropout rate, owing to gastrointestinal side effects, somnolence, and insomnia, as well as fast titration to higher dosages (Rascol et al., 2012).

Dipraglurant was studied in PD patients with moderate or severe LID in a phase IIa, randomized, placebo-controlled, double-blind, parallel-group trial. The primary goal was to establish safety and tolerability, and secondary goals were set to improve abnormal involuntary movements. A total of 46 (88.5%) dipraglurant-treated patients and 18 (75%) placebo patients experienced adverse events, with no significant changes in safety monitoring measures. Treatment with dipraglurant was linked with substantial effects on the mAIMS, but the durability of this initial impact is unknown, as there was no significant between-group difference at the study’s conclusion point (Tison et al., 2013). The impact of the mGluR-5 negative allosteric modulator dipraglurant on LID reduction without worsening of parkinsonism observed in one RCT suggests that this drug merits further research.

In Japan, istradefylline, an agonist of adenosine A_2A_ receptor, has been approved as a supplement to L-DOPA therapy for PD patients (Mizuno and Kondo, 2013). Its impact on locomotor disturbances and LID is debatable. In a placebo-controlled, double-blinded RCT containing 373 PD affected volunteers with motor abnormalities, it was found that by applying istradefylline along with L-DOPA, the off-time associated with L-DOPA therapy was significantly reduced, although dyskinesia was the most encountered adverse effect (Vorovenci, and Antonini, 2015). Preladenant, an adenosine A_2A_ receptor antagonist, was observed to be improving the on time associated with L-DOPA therapy, without changing the dyskinesia state in individuals with moderate to severe PD in a double-blinded, placebo-controlled RCT (Hauser et al., 2015). Preladenant was observed to be increasing the on time of L-DOPA, without severe dyskinesia in two double-blinded, placebo-controlled, phase III studies. However, the results obtained were not significant when compared with the placebo-treated group (Hauser et al., 2015). Caffeine is a commonly prescribed psychostimulant that works by blocking adenosine A2A and A1 receptors, which inhibits adenosine propagation (Fredholm et al., 1999). Caffeine causes a persistent and dose-dependent increase in DARPP-32 phosphorylation at Thr75, according to new research. Caffeine’s impact is greatest at 7.5 mg/kg, which also induces a sustained increase in motor activity. Caffeine’s capacity to enhance DARPP-32 phosphorylation at Thr75 is most likely mediated by adenosine A2A receptor inhibition as SCH 58261, a specific A2A receptor antagonist, similarly increases phosphoThr75 levels (Lindskog et al., 2002).

Clozapine, in numerous trials, including a placebo-controlled, double-blinded study, was observed to be decreasing the severity of LID (Durif et al., 2004; Seppi et al., 2011). Clozapine’s specific mechanism of action is unknown, but antagonistic binding to striatal DA receptor type 2 (D2) and serotonin type 2A receptors has been postulated (5-HT2A receptors). However, worries regarding possible side effects limit the use of clozapine for dyskinesia treatment. Insomnia, sialorrhea, asthenia, and possible severe toxicities, such as agranulocytosis in around 0.7 percent of patients, seizures, and myocarditis, were among the most prevalent side effects observed. Because of concerns regarding possible neutropenia, the constant neutrophil count was to be monitored for 12 months while on this drug (Pahwa et al., 2006; Seppi et al., 2011; Hack et al., 2014).

PET investigations in PD patients with LID show abnormalities in opioid receptors in the basal ganglia and elsewhere (Piccini et al., 1997). Furthermore, in rodent and primate models of LID, the expression and levels of opioid receptors are changed throughout the basal ganglia (Johansson et al., 2001). While there are numerous methodological variations across the trials, it appears that blocking opioid transmission in a fashion that is not selective for opioid receptor subtypes has no anti-dyskinetic effect in humans when used in conjunction with L-DOPA. This was proven in recent research in individuals with LID, where an intravenous infusion of the non-subtype-selective opioid antagonist naloxone at a dosage that had previously been shown to offer effective opioid transmission-blocking was unable to decrease opioid transmission (Fox et al., 2004).

Many other drugs which have shown promising results in the reduction of LID severity in animal models have failed to show their activity in clinical studies. Perampanel, topiramate, AQW051, rislenemdaz, vitamin D, and nitric oxide modulators (Lees et al., 2012; Kobylecki et al., 2014; Trenkwalder et al., 2016b; Herring et al., 2017; Habibi et al., 2018) are a few examples of it. Topiramate, an anti-epileptic drug, was even reported to be increasing the severity of dyskinesia in patients. Five patients were even withdrawn from the study due to increased dyskinesia (Kobylecki et al., 2014). However, more research is still required to fully understand these variations.

## 5 Problems in the Conversion of Preclinical Effectivity to Clinical Stage

The first challenge faced is the difference between the disease models used to evaluate effectivity in animals and humans. The animal models used for this purpose do not fully replicate the complex human neurodegenerative patterns and variations that can take place inside a human body. The gold standard preclinical model, that is, the MPTP-lesioned primate model of PD, is generally dopaminergic and non-progressive, but in the case of humans, there is the involvement of complex pathogenic pathways and progressive neurodegeneration. This fact can explain the lack of conversion of efficacious results from the preclinical to clinical phase (Fox and Brotchie, 2019).

Also, finding of equivalent dose between the preclinical to clinical phases is difficult. As an example, naftazone (CVXL0107), a glutamate inhibitor, showed to reduce LID in the MPTP-lesioned primate model of PD, but in a multicenter, crossover, double-blinded randomized control trial (DBRCT), it failed to alleviate the LID symptoms (Mattei et al., 1999; Brotchie et al., 2007; Corvol et al., 2019). The authors of this study have interpreted that the unsuccessful results are either due to a mismatch between L-DOPA/naftazone or due to inappropriate dosing. Famotidine, a selective H2 receptor antagonist, also failed to show clinical efficacy despite being effective in the preclinical studies. The reason behind this failure is poor BBB permeability (Johnston et al., 2010; Mestre et al., 2014). Another shortcoming of preclinical studies is that the animal models used cannot properly measure the tolerability in human subjects. Many drugs like topiramate and 5-HT1A antagonists have shown poor tolerability in human subjects, despite being efficacious in the preclinical studies (Goetz et al., 2017; Huot et al., 2017).

Measurement of LID severity in clinical trials is a difficult task. In preclinical studies, many objective rating scales are used to measure the LID severity, which are similar to objective rating scales used in clinical studies (UDYRS part III). This depends upon the patient’s perception of dyskinesia severity, and it provides no information about the level of disability. Along with that, lack of awareness of mild dyskinesia in PD is well documented (Hung et al., 2010). Thus, the results obtained from these scales are quite inaccurate, unreliable, and are subjected to recall bias (Stone et al., 2002; Papapetropoulos, 2012). However, some advanced technologies have been evaluated for the measurement of LID, and they were found to be producing more accurate and reliable results. Implementation of sensors to aid these technologies in measuring the severity and endpoints can also be an objective step that can produce more accurate results (Lopane et al., 2015; Ossig et al., 2016; Delrobaei et al., 2017).

The placebo effect in the evaluation of novel therapies alleviating LID is well observed in various studies. As an example, in a trial involving sarizotan, a 5HT1A agonist, the on time of LID was reduced by up to 1.5 h/day in the placebo-treated group, while the observed reduction in the LID on time was 2 h/day in the intervention group (Goetz et al., 2007; Goetz et al., 2008). The placebo effect is profound in the trials involving PD patients. The main underlying reason can be the reward effect associated with the DA pathology (Quattrone et al., 2018), that is, in blinded studies, after taking the placebo, there is a release of DA in the subject’s brain. These effects can lead to false-positive results and can mask the underlying effect of a novel therapy. Thus, understanding the placebo effect can guide the trial design of future clinical trials.

## 6 Conclusion

LID is a common and difficult condition to treat PD patients. Clinical phenomenology for LID varies, but the three types are high-dose dyskinesia, dressing off or off-period dyskinesia, and diphasic dyskinesia. The exact mechanism of action is not known for dyskinesia, but both presynaptic and postsynaptic methods are effective in the pathogenesis of LID, and the management of dyskinesia depends on the type of dyskinesia encountered and thereafter, is treated. Reduction of dyskinesia is dependent on the dose like peak-dose dyskinesia, while the addition of long-acting formulations is effective in wearing-off dyskinesia. Infusion and surgical procedures are working on all kinds of dyskinesia. Along with that, various other drug targets have also been identified like serotonergic and glutamatergic systems. Targeting these targets as an add-on therapy has proven to be very beneficial in the management of epilepsy. Chemical management with a neurosurgical approach is the future of the management of dyskinesia. Despite various advancements, the preclinical to clinical conversion rate is very low in the case of newly discovered drugs. Also, measuring the severity of dyskinesia has been challenging. More focus should be given to developing a progressive neurodegenerative animal model; a more unified LID measurement scale that does not depend on the patient’s perception is needed. Neurosurgical stimulation for primary mitigation, along with drug treatment as maintenance therapy, should be a rational approach going into the future. However, this combination is yet to be tested on humans.
