# Supplementary material for: Molecular Mechanisms and Therapeutic Strategies for Levodopa-Induced Dyskinesia in Parkinson’s Disease: A Perspective Through Preclinical and Clinical Evidence
Source: Front Pharmacol. 2022 Apr 7;13:805388. doi: 10.3389/fphar.2022.805388 (PMC9021725; doi:10.3389/fphar.2022.805388)
Supplement: Supplementary file 1 [file Table1.docx]

**Supplementary Table 1: Pre-clinical evidence in management of LID**

| **#** | **Compound /treatment (Dose, Route)** | **Class of compound** | **Experimental**  **model** | **Animal/ cell lines** | **Outcomes** | **Ref** |
| --- | --- | --- | --- | --- | --- | --- |
| **Dopaminergic system** | | | | | | |
| 1 | Chronic low lode of cabergoline (dose ranging from 0.015 to 0.035 mg/kg; s.c.), for 1 month | Dopamine agonist | MPTP induced PD model followed by onset of LID by L-DOPA | Female cynomolgus (*Macaca fascicularis*) monkeys | Inhibition of LID development, without affecting the anti-PD effects of L-DOPA | Bélanger et al., 2003 |
| 2 | Safinamide (15 mg/kg) | MAO-B inhibitor | 6-OHDA induced PD followed by L-DOPA induced dyskinesia | Female Sprague Dawley rats (150–175 g) | Alleviation of LID, and striatal glutamate modulating activity | Gardoni et al., 2018 |
| **3** | Selegiline (10 mg/kg; s.c.) | MAO-B inhibitor | 6-hydroxydopamine induced PD followed by induction of LID by using daily dose of L-DOPA | Male Sprague Dawley rats (7 weeks old) | Effective in increasing the on-time of L-DOPA without affecting the dyskinesia | Tsunekawa et al., 2018 |
| 4 | Levodopa/Benserazide PLGA microsphere | Water in oil emulsion to maintain constant plasma concentration of levodopa | 6-OHDA induced PD model | Male Sprague Dawley rats (4-9 months, 200-250 g) | Severity of LID decreased when compared with standard formulation | Wang et al., 2019 |
| **5** | L-dopa (15 mg/kg, i.p.) and Benserazide (3.75 mg/kg, i.p.) along with Upregulation of β-arrestin2 via rAAV vector | ----------- | 6-OHDA induced PD followed by L-DOPA induced dyskinesia | Male Sprague Dawley rats (182-250 g) | Reduction of LID via inhibition of G protein-dependent D1R and phosphor-DARPP32/ERK signaling | Zhang et al., 2019 |
| **6** | Poly (L-DOPA)-based self-assembled nanodrug (Nano^DOPA^) | L-DOPA precursor | MPTP induced PD | Male C57BL/6 J mice (15 week) old | Significant improvement of PD symptoms compared with only l-DOPA treatment, and no dyskinesia was observed | Vong et al., 2020 |
| **7** | Sustained Cabergoline administration (0.125 to 0.185 mg/kg, s.c) | Dopamine agonist | MPTP induced PD model followed by onset of LID by L-DOPA | Female Cynomolgus (*Macaca fascicularis*) monkeys (3.25–4.80 kg) | Induction of dyskinesia initially then gradual Reduction of LID without affecting the antiparkinsonian effect of L-DOPA | Hadj et al., 2000 |
| **Non- dopaminergic therapy** | | | | | | |
| 01 | ST1535 (20 mg/kg, p.o.) for 8 weeks | Adenosine A2 receptor antagonist | MPTP induced PD model followed by onset of LID by L-DOPA | Male and female Marmosets (365-437 g) | Effective as an antiparkinsonian agent and helps in reduction of L-DOPA dose required in the treatment of PD. Thus, can reduce the LID development | Rose et al., 2006 |
| 02 | Caffeine (3 and 15 mg/kg, i.p.) for 21 days | Adenosine A2 receptor antagonist | 6-OHDA induced PD followed by L-DOPA induced dyskinesia | Wild type mice (24 M and 12 F) | Alleviation of LID symptoms via blockage of A_1_ or A_2A_ receptors | Xiao et al., 2011 |
| 03 | Flibanserin (10 mg/kg; i.p) | 5-HT1A receptors, a weak partial agonist at D4 receptors, and an antagonist at 5-HT2A-receptors | Reserpine induced PD followed by L-DOPA or quinelorane induced dyskinesia | 20 C57BL/6 wildtype (10 males, 10 females) and 20 RGS9 knock-out mice (10 male, 10 female), 3–5 months old | Decrease in levodopa induced dystonia, i.e., antidyskinetic effect | Strecker et al., 2012 |
| 04 | SCH 412348 (3 mg/kg, i.p.) for 22 days | Adenosine A2 receptor antagonist | 6-OHDA induced PD followed by L-DOPA induced dyskinesia | Male Sprague Dawley rats (350-400g) | Adenosine A2 receptor antagonists do not alleviate LID when co-administered with L-DOPA | Jones et al., 2013 |
| 05 | Istradefylline (10 mg/kg, p.o.) for 28 days | Adenosine A2 receptor antagonist | MPTP induced PD model followed by onset of LID by L-DOPA | Male and female Marmosets (285-420 g) | Effective in improving motor complications associated with PD without provoking dyskinesia | Uchida et al., 2015 |
| 06 | Combination of Eltoprazine (1 mg/kg) and Preladenant (5 mg/kg) | Eltoprazine- 5-HT_1A/B_ receptor agonist  Preladenant-selective adenosine A_2a_ receptor antagonist | MPTP induced PD model followed by onset of LID by L-DOPA | Captive bred female monkeys (*Macaca fascicularis*, 3.7 kg, 9 years) | Short term reduction of dyskinesia but not able to maintain the reduced dyskinesia expression for longer period. | Ko et al., 2017 |
| 07 | Blackberry (*Morus nigra*) (5, 10, and 15 mL/kg; oral) | Principal API- anthocyanin which is a flavonoid | MPTP induced PD model followed by onset of LID by L-DOPA | male BALB/c mice (25–30 g) | 10, 15 ml/kg doses were most effective in reducing AIMS score i.e., reducing LID. | Fahimi and Jahromy, 2018 |
| 08 | Lactomorphin (MMP-2200) (0.8 mg/kg, s.c.) | Mu-delta opioid receptor agonist | 6-OHDA induced PD followed by L-DOPA induced dyskinesia | Male Sprague Dawley rats (250 g) | Antiparkinsonian activity with reduced LAO AIMs scores | Flores et al., 2018 |
| 09 | MK-801 in combination with Lactomorphin | NMDA receptor antagonist | 6-OHDA induced PD followed by L-DOPA induced dyskinesia | Male Sprague Dawley rats (250 g) | Reduction of L-DOPA induced AIMs, without stimulating PD | Flores et al., 2018 |
| 10 | LY-354,740 (eglumegad) (1 mg/kg) | mGlu_2/3_ orthosteric agonist | 6-OHDA lesion induced PD followed by L-DOPA induced dyskinesia | female Sprague-Dawley rats (250–275 g) and Marmosets (300-450 g) | Reduction of dyskinesia and psychosis like behavior. Also, potentiation of the therapeutic benefit of L-DOPA | Frouni et al., 2019 |
| 11 | Memantine (intra striatum injection) | Non-competitive NMDA receptor antagonist | 6-OHDA- induced hemi parkinsonian mouse receiving daily dose of L-DOPA | Adult male C57BL/6 (8–9) weeks | continuous infusion is useful in Alleviation of dyskinesia | Ogawa et al., 2019 |
| 12 | ZNS (26 mg/kg; s.c.) | Anticonvulsants | 6-OHDA induced PD followed by L-DOPA induced dyskinesia | Female Sprague-Dawley rats (220–250 g) | Alleviation of LID via inhibited upregulation of adenosine A2A and endocannabinoids CB1 receptors | Ogawa et al., 2019 |
| 13 | Cyprodime, ADC-02520849, ADC-02265510 (15 µg/µl; Intra cerebral injection) | Opioid agonist | MPTP Induced PD followed by L-DOPA induced dyskinesia | Macaque monkeys | Opioid receptor agonists alleviate LID symptoms and decrease the LID severity scores. | Bezard et al., 2020 |
| 14 | Rapamycin (2 mg/kg; i.p) | Macrolide antibiotics | 6-OHDA induced PD followed by L-DOPA induced dyskinesia | Male Wistar rats | Reduction of the magnitude of LID by preventing the loss of striatal bidirectional plasticity via inhibition of mTORC1 signaling | Calabrese et al., 2020 |
| 15 | fasudil (10 and 40 mg/kg) | ROCK inhibitor | 6-OHDA induced PD followed by L-DOPA induced dyskinesia | Adult female Sprague Dawley rats (225-250 g) | Reduction of development of LID without affecting the therapeutic antiparkinsonian effect of L-DOPA | Lopez-Lopez et al., 2020 |
| 16 | vilazodone (10 mg/kg; i.p) | SSRI and 5-HT1A partial agonist | 6-OHDA induced PD followed by L-DOPA induced dyskinesia | Adult male Sprague Dawley rats (225–249 g) | Suppression of L-Dopa induced Dyskinesia without affecting the anti PD effect of L-DOPA and inhibition of gene regulation which are critical for LID. | Altwal et al., 2020 |
| 17 | Δ^9^-THCV (2 mg/kg; i.p) | antioxidant and CB_2_ receptor agonist | L- DOPA induced dyskinesia in genetic dopamine deficient mouse | Pitx3^ak^ mutant male mice (4–6 months, 24–30 g) | Delay the occurrence and reduces the magnitude of LID | Espadas et al., 2020 |
| 18 | Ketamine (20 mg/kg; i.p) | General anesthetics | 6-OHDA induced PD followed by L-DOPA induced dyskinesia | Adult male, Sprague-Dawley rats (225 g) | Anti-dyskinetic activity via modulation of neurotrophic factor-release in the striatum, followed by ERK1/2 and mTOR signaling pathway activation | Bartlett et al., 2020 |
| 19 | PT320 (SR formulation of exendin-4) (PLGA microspheres (100 mg/kg, oral) | GLP-1R agonist | 6-OHDA induced PD followed by L-DOPA induced dyskinesia | Adult male Sprague–Dawley rats | Alleviation of dyskinesia via reduction of AIM scores | Yu et al., 2020 |
| 20 | MR1916 (0.03‒0.3 mg/kg; po) | Phosphodiesterase 10A (PDE10A) inhibitor | 6-OHDA induced PD followed by L-DOPA induced dyskinesia | Male Sprague–Dawley rats | Dose dependent reduction of LID without effecting the anti- parkinsonian effects of L-DOPA | Arakawa et al., 2020 |
| 21 | Thalidomide and 3,6'-dithio thalidomide (70 mg/kg) | Immuno-modulatory agent | 6-hydroxydopamine induced parkinsonian mouse receiving daily dose of L-DOPA | Sprague-Dawley rats (270–300 g) | Alleviation of dyskinesia by reducing neuroinflammation via reduction of TNF-α and resorting expression of IL-10. | Boi et al., 2021 |

Δ^9^-THCV: Delta-9- tetrahydrocannabivarin; ZNS: Zonisamide; PLGA: Poly Lactic-co-Glycolic Acid; AIMS: Abnormal involuntary movement score; mTORC1: mammalian target of rapamycin complex 1; ROCK: Rho kinase; LAO AIMs: limb, axial, and oral anormal involuntary movements.
