# Supplementary material for: Molecular Mechanisms and Therapeutic Strategies for Levodopa-Induced Dyskinesia in Parkinson’s Disease: A Perspective Through Preclinical and Clinical Evidence
Source: Front Pharmacol. 2022 Apr 7;13:805388. doi: 10.3389/fphar.2022.805388 (PMC9021725; doi:10.3389/fphar.2022.805388)
Supplement: Supplementary file 2 [file Table2.docx]

**Supplementary Table 2: Clinical evidence regarding the management of LID**

| **#** | **Intervention** | **Class of treatment** | **Population** | **experimental design** | | **Outcomes** | **Ref** |
| --- | --- | --- | --- | --- | --- | --- | --- |
| **Dopamine therapy** | | | | | | | |
| 01 | L-Dopa standard (150, 300, 600 mg/day) | Dopamine precursor | 361 PD patients suffering from LID | 40-week, dose-response L-dopa placebo controlled RCT | Increased parkinsonism in placebo group, increased incidence of LID, fluctuations in L-DOPA levels at higher dose | | Fahn, 2006 |
| 02 | Rapid onset formulation of L-DOPA | Dopamine precursor | 74 PD patients suffering from LID | 4 + 8 weeks, L-dopa vs. melevodopa afternoon dose | No effect on LID but there is an increased ONN time of L-DOPA | | Stocchi et al., 2007 |
| 03 | Pramipexole | Dopamine agonist | 354 PD patients suffering from LID | Double-blinded, placebo-controlled, open labeled, Phase III study; 32 weeks | Improved parkinsonism, decreased UPDRS II–IV scores and increased LID | | Möller et al., 2005 |
| 04 | Pramipexole (0.5 mg/day in 1^st^ week, 0..75 mg/day in 2^nd^ week, 1.0 mg/day in 3^rd^ week, 1.5 mg/day in 4^th^ week; after dinner and breakfast) | Dopamine agonist | 34 PD patients suffering from dyskinesia | 24 weeks, multicenter, randomized, open-label, parallel-group comparison study | Alleviation of LID due to its D3 receptor agonistic property which balances the D1 receptor super sensitivity | | Utsumi et al., 2013 |
| 05 | LCIG infusion (16 hours of daily infusion for 12 months) | Combination of Dopamine precursor and Dopa decarboxylase inhibitor | 10 PD patients (5 F, 10 M) with LID and on dopamine agonist medication | 12 months, Open labeled, prospective study | Improvement of motor functions, quality of life and increase in daily ONN period. Only side effect encountered was peripheral neuropathy, but it can be reduced by proper supplementation. Cannot be practiced for vitamin B12 and B6 deficit patients | | Chang et al., 2016 |
| 06 | ADS-5102 (amantadine extended-release capsules, 274 mg during bedtime) | Dopamine agonist | 189 PD patients were screened, and 126 patients were randomized | 14 months, double-blinded, placebo-controlled, RCT | Reduction of dyskinesia scores and reduced OFF time associated with L-DOPA treatment | | Pahwa et al., 2017 |
| 07 | amantadine extended-release capsules (274 mg) | Dopamine agonist | 114 PD patients enrolled and finally 77 patients are randomized | A phase III, randomized, double‐blind, placebo‐controlled, multi-center study | Reduction of dyskinesia and OFF time associated with L-DOPA | | Oertel et al., 2017 |
| 08 | *Mucuna pruriens* (MP) roasted seeds (high dose- 17.5 mg/kg, low dose- 12.5 mg/kg) | Leguminous plant containing levodopa | 18 patients with advanced PD | Randomized, placebo-controlled phase II clinical trial, patients were evaluated 0 to 180 minutes after taking the MP doses | Greater motor improvements with high dose of MP when compared to levodopa and placebo treated groups, longer ONN period and reduction of dyskinesia. Also, with MP administration fewer side effects are encountered | | Cilia et al., 2017 |
| 09 | LCIG | Dopamine precursor and Dopa decarboxylase inhibitor | 375 PD patients registered in GLORIA registry. Amongst them 118 having <4 h/day of dyskinesia and 139 patients with ≥4 h/day dyskinesia at baseline | 24 months, phase II study, with concomitant PD medication allowance after principal investigator’s approval | Mean dyskinesia duration gets significantly decreased by 3.5 h for patients having ≥4 h baseline dyskinesia but on the other hand dyskinesia duration increases by 1.6 h for patients with <4 h baseline dyskinesia. L-Dopa dose gets increased, but LID severity and associated pain gets decreased | | Poewe et al., 2018 |
| 10 | Gocovri® (amantadine) extended-release capsules (274 mg once daily during daytime) | Dopamine agonist | 223 PD patients having LID | 2 year, open-label trial | Reduction of LID (dyskinesia and OFF time) along with long term safety, tolerability, and efficacy | | Tanner et al., 2020 |
| **Non- dopaminergic therapy** | | | | | | | |
| 01 | Fipamezole (30, 60, and 90 mg) | selective α2-adrenergic receptor antagonist | 179 PD patients with LID | 28-days, Double-blind, randomized, placebo-controlled, multi centered, dose-escalating study | 90 mg fipamezole reduced LID (mean, 95% CI, LID rating improvement vs placebo -1.9 [0.0 to -3.8; p = 0.047]) without exacerbating parkinsonism | | Lewitt et al., 2012 |
| 02 | Perampanel (2 mg and 4 mg) | selective, noncompetitive α-amino-3-hydroxy-5-methyl-4-isoxazole-propionic acid receptor antagonist | 1,514 PD patients suffering from LID | multicenter (two centers) randomized, double-blind, placebo-controlled, parallel-group phase III studies, one study was for 30 weeks and other one was for 20 weeks) | No significant improvement od LID symptoms when compared with placebo, and also no effect on duration or disability of LID. | | Lees et al., 2011 |
| 03 | AFQ056 (at doses of 20, 50, 100, 150, or 200 mg daily) | selective metabotropic glutamate receptor 5 antagonist | PD patients with moderate to severe LID | 13-week, double-blinded, placebo-controlled, RCT | Significant improvement of Unified Parkinson's Disease Rating Scale part IV score, without worsening the PD symptoms | | Stocchi et al., 2013 |
| 04 | Topiramate (100 mg/day) | Anti- epileptic | 15 PD patients with stable LID | 4 weeks, double-blind, placebo-controlled, crossover RCT, followed by a 2 weeks of assessment period | Increased dyskinesia severity when compared to placebo treatment and 5 patients were withdrawn from the study due to adverse effects | | Kobylecki et al., 2014 |
| 05 | low frequency (1Hz) rTMS | rTMS induced stimulation of SMA (supplementary motor area) of brain | 17 PD patients suffering from LID | 10 days, randomized, sham (placebo) controlled study | Reduction of dyskinesia lasting for 24 hours, without reducing the PD associated motor complications. Long term observation and experiment on larger population is needed | | Sayın et al., 2014 |
| 06 | Eltoprazine (2.5, 5 and 7.5 mg, oral) | 5-HT1A and 5-HT1B receptor agonist | 22 PD patients suffering from LID | A double-blinded, randomized, placebo-controlled and dose-finding phase I/IIa study | 5 mg and 7.5 mg doses showed significant reduction in dyskinesia, without altering the normal motor responses of L-DOPA. All the doses were well tolerated | | Svenningsson et al., 2015 |
| 07 | memantine (20 mg) | Glutamate antagonist | 21PD patients suffering from LID | 3 weeks, randomized, double-blind and placebo-controlled, crossover clinical trial | Well tolerated, no worsening of PD motor symptoms, and reduction of LID | | Wictorin et al., 2016 |
| 08 | Dipraglurant (gradually escalated dose from 50 mg once daily to 100 mg 3 times a day) | metabotropic glutamate receptor 5-negative allosteric modulator | 76 PD patients with moderate to severe LID | 4-week, double-blinded, placebo-controlled, randomized (2:1), parallel-group, multicenter dose-escalation, phase 2A clinical trial | Significant reduction of peak dose dyskinesia, no worsening of parkinsonism, rapid absorption (t_max_ = 1 hour) and C_max_ obtained = 1844 ng/mL on day 28. | | Tison et al., 2016 |
| 09 | AQW051 (10 mg and 50 mg once daily) | nicotinic acetylcholine receptor α7 agonist | 71idiopathic PD patients with moderate to severe LID | Randomized, double-blinded, placebo-controlled, phase II study | No significant improvement of AIMs or UPDRS-III scores by day 28 and side effects encountered was dyskinesia, fatigue, nausea, and falls | | Trenkwalder et al., 2016 |
| 10 | Dextromethorphan + quinidine (45 mg +10 mg; twice daily) | sigma-1 receptor-agonist + glutamatergic /monoaminergic modulator | 13 PD patients suffering from LID | 2 week, double-blind, crossover design, placebo controlled RCT with intervening 2-week washout | Significant decrease in the mean dyskinesia scores with no effect on PD motor scores | | Fox et al., 2017 |
| 11 | MK-0657 (7 mg) | NR2B selective NMDA receptor antagonist | 22 PD patients suffering from LID | A double-blinded, single-dose, placebo controlled, 2-period crossover, RCT study | A single dose of MK-0657 is unable to alleviate the LID symptoms | | Herring et al., 2017 |
| 12 | Vitamin D (1000 IU/d) | Vitamin | 120 PD patients | 3 months, double blinded, placebo controlled, RCT | No effect on PD and dyskinesia scores i.e., Vitamin D has no effect on PD or dyskinesia | | Habibi et al., 2018 |
| 13 | Repetitive transcranial stimulation | Neurosurgical procedure | 17 Parkinson disease patients with peak-of-dose dyskinesia | 30 min of Repetitive transcranial stimulation on pre-supplementary motor area followed by 200 mg levodopa tablets intake, then analysis of brain by fMRI | 1 Hz rTMS delays the onset and reduces the severity of LID | | Lohse et al., 2020 |
| 14 | Cerebellar continuous theta burst stimulation (cTBS) (after the levodopa administration) | Neurosurgical procedure | 11 PD patients suffering from PD | 125% of usual levodopa dose administration then after that application of cTBS | Reduction of LID which is associated with reduction of BNDF Val66Met polymorphism | | Sanna et al., 2019 |
| 15 | 5-Hydroxytryptophan (30mg daily) | Antidepressant/serotonin precursor | 12 PD patients diagnosed with LID | Placebo controlled RCT with cross over design, for a duration of 4 week | Improvement of LID measured by UDysRS and UPDRS-IV scores | | Meloniet al., 2020 |
| 16 | Mavoglurant (AFQ056) | selective metabotropic glutamate receptor-5 (mGluR5) inhibitor | levodopa-induced dyskinesia (LID) in patients with Parkinson's Disease (PD) | A meta-analysis involving 6 placebo controlled RCTs depicting the effect of Mavoglurant in 485 PD patients | No significant difference in ONN time, OFF time, Lang-Fahn activities of daily living dyskinesia scale, UPDRS-III and UPDRS-IV scores when compared to the placebo treated groups i.e., no effect of Mavoglurant on PD and LID | | Negida et al., 2021 |
|  |  |  |  |  |  | |  |

RCT: Randomized control trial; LCIG: Levodopa–carbidopa intestinal gel; AIMs: Abnormal Involuntary Movements score; UPDRS: unified Parkinson's disease rating scale, rTMS: repetitive transcranial magnetic stimulation; LCIG: intraduodenal levodopa-carbidopa intestinal gel;
